# 超高效液相色谱-串联质谱法测定人尿中12种典型个人护理品

**DOI:** 10.3724/SP.J.1123.2022.05032

**Published:** 2023-04-08

**Authors:** Linxue HAN, Xu ZHANG, Xiaojian HU, Haijing ZHANG, Tian QIU, Xiao LIN, Ying ZHU

**Affiliations:** 中国疾病预防控制中心环境与健康相关产品安全所, 中国疾病预防控制中心环境与人群健康重点实验室, 北京 100021; China CDC Key Laboratory of Environment and Population Health, National Institute of Environmental Health, Chinese Center for Disease Control and Prevention, Beijing 100021, China

**Keywords:** 固相萃取, 超高效液相色谱-串联质谱, 个人护理品, 尿液, 生物监测, solid phase extraction (SPE), ultra performance liquid chromatography-tandem mass spectrometry (UPLC-MS/MS), personal care products (PCPs), urine, biomonitoring

## Abstract

研究建立了一种超高效液相色谱-串联质谱法(UPLC-MS/MS)同时测定人尿中12种典型个人护理品(PCPs)的分析方法,其中包括5种对羟基苯甲酸酯类防腐剂(PBs)、5种二苯酮类紫外吸收剂(BPs)和2种抗菌剂。1 mL尿样中加入*β*-葡萄糖醛酸酶-乙酸铵缓冲溶液和混合内标工作液置于37 ℃水浴酶解过夜(≥16 h)。样品采用Oasis HLB固相萃取柱富集净化,在乙腈-水流动相体系下采用Acquity BEH C_18_色谱柱进行色谱分离,负离子电喷雾(ESI^-^)多反应监测(MRM)模式检测,稳定同位素内标法定量。实验通过优化仪器质谱参数、流动相和色谱柱等色谱条件以及酶解和固相萃取柱等前处理条件,获得了最佳实验结果。在优化条件下,羟苯甲酯(MeP)和二苯酮-3(BP-3)在4.00~800 μg/L,三氯生(TCS)在5.00~200 μg/L,其余9种PCPs在1.00~200 μg/L范围内线性关系良好,相关系数大于0.999,方法检出限(MDL)为0.06~1.09 μg/L,方法定量限(MQL)为0.08~3.63 μg/L。12种分析物的加标回收率为89.5%~111.8%,日内精密度为3.7%~8.9%,日间精密度为2.0%~10.6%。采用稳定同位素内标法校正后12种分析物的基质效应为91.9%~110.1%。该方法成功应用于127份实际尿液样品的测定。结果表明,除羟苯苄酯(BzP)和二苯酮-8(BP-8)未检出外,其余10种典型PCPs均可检出,总体检出率为1.7%~99.7%,其中MeP、羟苯乙酯(EtP)和羟苯丙酯(PrP)检出率和浓度水平处于较高水平。该方法简便灵敏,可为人群尿液样本PCPs的生物监测工作提供可靠的技术支持。

## Abstract (en)

A rapid and sensitive method based on ultra performance liquid chromatography-tandem mass spectrometry (UPLC-MS/MS) was developed for the simultaneous determination of 12 typical personal care products (PCPs) in human urine. These PCPs included five paraben preservatives (PBs), five benzophenone UV absorbers (BPs), and two antibacterial agents. Accordingly, 1 mL of the urine sample was mixed with 500 μL of *β*-glucuronidase-ammonium acetate buffer solution (enzymatic activities are 500 units/mL) and 75 μL of a mixed internal standard working solution (internal standard contents are 7.5 ng), followed by enzymatic hydrolysis overnight (≥16 h) at 37 ℃ in a water bath. The 12 targeted analytes were enriched and cleaned up using an Oasis HLB solid phase extraction column. Separation was performed on an Acquity BEH C_18_ column (100 mm×2.1 mm, 1.7 μm) using an acetonitrile-water system as the mobile phase, in negative electrospray ionization (ESI^-^) multiple reaction monitoring (MRM) mode, for target detection and stable isotope internal standard quantification. The optimal MS conditions were established by optimizing the instrument parameters and comparing two analytical columns (Acquity BEH C_18_ and Acquity UPLC HSS T_3_) as well as different types of mobile phases (methanol or acetonitrile as the organic phase) to achieve better chromatographic separation. In order to obtain higher enzymatic and extraction efficiency, different enzymatic conditions, solid phase extraction columns, and elution conditions were investigated. The final results showed that methyl parabens (MeP), benzophenone-3 (BP-3), and triclosan (TCS) showed good linearities in the ranges of 4.00-800, 4.00-800 and 5.00-200 μg/L, respectively, the other targeted compounds showed good linearities in the ranges of 1.00-200 μg/L. The correlation coefficients were all greater than 0.999. The method detection limits (MDLs) were in the range of 0.06-1.09 μg/L, and the method quantification limits (MQLs) ranged from 0.08 to 3.63 μg/L. At three spiked levels, the average recoveries of the 12 targeted analytes ranged from 89.5% to 111.8%. The intra-day and inter-day precisions were 3.7%-8.9% and 2.0%-10.6%, respectively. The results of the matrix effect assessment showed that MeP, ethyl paraben (EtP), and benzophenone-2 (BP-2) exhibited strong matrix effects (26.7%-103.8%); propyl paraben (PrP) exhibited moderate matrix effects (79.2%-112.0%); and the other eight target analytes exhibited weak matrix effects (83.3%-113.8%). The matrix effects of the 12 targeted analytes after correction using the stable isotopic internal standard method ranged from 91.9% to 110.1%. The developed method was successfully applied to the determination of the 12 PCPs in 127 urine samples. Ten typical PCPs were detected, with the overall detection rates ranging from 1.7% to 99.7%, except for benzyl paraben (BzP) and benzophenone-8 (BP-8). The results revealed that the population in this area was widely exposed to PCPs, especially MeP, EtP and PrP; the detection rates and concentrations of these PCPs were found to be very high. Our analytical method is simple and sensitive, and it is expected to be an effective tool for biomonitoring PCPs in human urine samples as part of environmental health studies.

个人护理品(PCPs)在1999年被Daughton提出,主要包括防腐剂、紫外吸收剂、抗菌剂、芳香剂和驱虫剂等^[1]^,其作为功能性助剂广泛应用于口红、防晒霜和洗涤剂等日用产品中。PCPs可随产品应用进入到环境介质,目前包括地表水、地下水、污水处理厂的进出水、土壤、大气在内的多种环境介质,以及植物组织、水生动物脂肪组织中均存在该类物质的残留^[2⇓⇓-5]^, PCPs的广泛、大量使用及其在环境中的普遍存在导致该类物质在人体内的长周期暴露。如Zhang等^[6]^在中国广州室内灰尘和人体尿液配对样品中广泛检测到PCPs,主要包括对羟基苯甲酸酯(PB)类防腐剂和二苯酮(BP)类紫外吸收剂,其检出率均高于70%,其中羟苯甲酯(MeP)在人尿中的检出率可达100%。总PB水平在室内灰尘和人尿中分别为1970~19600 ng/g和63.2~1041 μg/L,总BP水平为27.7~149 ng/g和1.77~21.0 μg/L。Perez等^[7]^研究表明,我国珠江地区土壤样品中MeP、羟苯乙酯(EtP)和羟苯丁酯(BuP)均有检出,且其最高含量达8.04、1.23和1.02 ng/g。此外,流行病学结果提示,过敏、哮喘、狼疮等免疫系统疾病和乳腺癌、子宫内膜异位症等生殖系统疾病可能与PCPs暴露相关^[8⇓⇓⇓-12]^。动物实验证实,PCPs在一定程度上具有与持久性有机污染物相类似的“持久性、生物富集性和生物毒性”,对模式动物具有遗传毒性、生殖毒性、内分泌干扰效应和明显的雌激素活性^[13⇓⇓-16]^,是潜在的内分泌干扰物^[17]^。因此,目前多个国家已限制了部分PCPs在食品和化妆品中的最大允许使用量,如欧盟、澳大利亚、韩国和日本对化妆品中BP的限值分别为6%、10%、5%和5%^[18]^。我国食品添加剂使用卫生标准(GB 2760-2014)^[19]^规定,PB用量为0.012~0.5 g/kg(以酸计),我国《化妆品安全技术规范》(2015年版)^[20]^中规定PB的使用上限为单一酯0.4%(以酸计)、混合酯0.8%(以酸计)等。

鉴于PCPs存在的多种毒性效应,其暴露人群的健康风险受到广泛关注。人群生物监测可以准确地反映人群暴露水平与特征,成为开展环境污染物健康影响研究的“金标准”。当前,美国和加拿大等发达国家均已对本国居民开展了该类物质的生物监测工作^[21,22]^,涉及的监测指标包括MeP、EtP、羟苯丙酯(PrP)、BuP、二苯酮-3(BP-3)、三氯生(TCS)和三氯卡班(TCC)等。采用的检测方法主要包括气相色谱-质谱联用技术(GC-MS)^[23]^和液相色谱-质谱联用技术(LC-MS)^[24]^。其中,GC-MS需要进行衍生化,过程繁琐耗时,对实验人员和环境不友好,不适合大样本检测;国外生物监测使用较多的是在线固相萃取-高效液相色谱-串联质谱法(Online SPE-HPLC-MS/MS)^[25]^,但在国内普及性不高,不利于方法推广。国内除采用上述检测方法以外,高效液相色谱-紫外检测器(HPLC-UV)和高效液相色谱-二极管阵列检测器(HPLC-DAD)等方法也常应用于尿液中PCPs的检测,但该类方法灵敏度不高^[26]^。

基于此,本研究最终选用超高效液相色谱-串联质谱法(UPLC-MS/MS),前处理采用固相萃取方法。检测指标方面,在美国生物监测指标基础上,增加了部分防腐剂和紫外线吸收剂,共纳入包括5种PB类防腐剂、5种BP类紫外吸收剂和2种抗菌剂(TCS、TCC)在内的12种典型PCPs。该方法具有操作简便、定量准确、重复性好且所需样本量少、可有效推广等优点,并成功应用于实际人群尿液样品的检测。

## 1 实验部分

### 1.1 仪器、试剂与材料

I-Class Plus型超高效液相色谱-三重四极杆质谱仪(TQ-XS)、Oasis HLB固相萃取柱(美国Waters公司); 5910R型低温离心机(德国Eppendorf公司);恒温水浴摇床(中国莱伯泰科仪器股份有限公司); 24孔固相萃取装置(美国Supelco公司); AP-250D电子天平(美国Ohaus公司); Milli-IQ-7005纯水机(美国Millipore公司); MULTIVAP-48氮吹仪(美国Organomation公司)。

MeP、EtP、PrP、BuP和羟苯苄酯(BzP)(质量浓度为1 mg/mL,纯度>98%)购自美国Cambridge Isotope Laboratories公司;BP-3、TCC(质量浓度为10 mg/mL,纯度>98%)和罗曼蜗牛(*Helix pomatia*)*β*-葡萄糖醛酸酶(Type H-2, >85000 units/mL)购自美国Sigma Aldrich公司;二苯酮-1(BP-1)、二苯酮-2(BP-2)、二苯酮-8(BP-8)、4-羟基二苯酮(4-OHBP)、TCS及12种稳定同位素内标(MeP-^13^C_6_、EtP-^13^C_6_、PrP-^13^C_6_、BuP-^13^C_6_、BzP-D_7_、BP-1-D_5_、BP-2-D_4_、BP-3-^13^C_6_、BP-8-D_3_、4-OHBP-D_4_、TCC-D_4_、TCS-^13^C_12_)购自加拿大Toronto Research Chemicals公司,纯度均>97%。甲醇(LC-MS级)和乙腈(LC-MS级)购自德国Merck公司;乙酸和乙酸铵(MS级)购自美国Thermo Fisher公司;美国国家标准与技术研究院标准参考物质3672(NIST SRM 3672)。

### 1.2 实验条件

#### 1.2.1 色谱条件

色谱柱:Waters Acquity BEH C_18_色谱柱(100 mm×2.1 mm, 1.7 μm);流动相A:水;流动相B:乙腈;进样量:5 μL;柱温:40 ℃;流速:0.3 mL/min。梯度洗脱程序见表1。

**表1:** 梯度洗脱程序

Time/min	φ(Water)/%	φ(Acetonitrile)/%
0	80.0	20.0
2.00	80.0	20.0
16.00	20.0	80.0
16.30	20.0	80.0

#### 1.2.2 质谱条件

离子源:电喷雾电离(ESI)源;电离方式:负离子模式(ESI^-^);扫描模式:多离子反应监测(MRM)模式;毛细管电压:2.46 kV;离子源温度:150 ℃;脱溶剂温度:500 ℃。12种分析物及其稳定同位素内标的详细质谱参数见表2。

**表2:** 12种分析物及其稳定同位素内标的质谱参数

Type	Compound	Retention time/min	Parent ion (m/z)	Daughter ion (m/z)	Cone voltage/V	Collision energy/eV
Parabens	methyl paraben (MeP)	4.44	151.02	91.95^*^	-8	-20
				135.97	-8	-14
	ethyl paraben (EtP)	6.55	165.03	92.00^*^	-24	-20
				137.00	-24	-20
	propyl paraben (PrP)	8.36	179.07	92.07^*^	-16	-20
				136.00	-16	-14
	butyl paraben (BuP)	9.91	193.00	92.07^*^	-36	-22
				136.32	-36	-16
	benzyl paraben (BzP)	9.99	226.98	92.00^*^	-28	-14
				136.06	-28	-24
	MeP-^13^C_6_	4.44	157.07	97.78	-2	-18
	EtP-^13^C_6_	6.54	171.10	98.03	-8	-20
	PrP-^13^C_6_	8.33	185.06	97.84	-10	-22
	BuP-^13^C_6_	9.90	199.08	97.90	-16	-22
	BzP-D_7_	9.94	234.13	91.77	-8	-26
Benzophenones	benzophenone-1 (BP-1)	9.14	212.97	135.04^*^	-16	-24
				91.03	-16	-20
	benzophenone-2 (BP-2)	5.66	245.02	135.03^*^	-10	-28
				91.03	-10	-16
	benzophenone-3 (BP-3)	12.17	227.14	211.16^*^	-8	-34
				167.02	-8	-16
	benzophenone-8 (BP-8)	10.21	243.87	93.10^*^	-20	-22
				123.51	-20	-16
	4-hydroxy benzophenone (4-OHBP)	7.87	196.91	91.95^*^	-10	-28
				120.07	-10	-24
	BP-1-D_5_	9.13	218.09	134.87	-36	-20
	BP-2-D_4_	5.64	249.08	136.82	-4	-16
	BP-3-^13^C_12_	12.15	233.10	217.20	-44	-16
	BP-8-D_3_	10.19	246.10	125.94	-20	-18
	4-OHBP-D_4_	7.86	200.95	95.82	-6	-30
Antimicrobials	triclocarban (TCC)	13.77	313.01	160.00^*^	-1	-12
				126.10	-12	-28
	triclosan (TCS)	14.01	286.80	35.00^*^	-20	-10
			289.00	35.00	-20	-12
	TCC-D_4_	13.76	317.00	160.00	-12	-12
	TCS-^13^C_12_	13.99	299.00	34.90	-20	-5

* Quantitative ion.

### 1.3 溶液配制

乙酸铵-酶缓冲溶液:准确移取235 μL *β*-葡萄糖醛酸酶溶于20 mL乙酸铵缓冲溶液(pH=5.0)。

混合标准溶液:准确移取质量浓度为1 mg/mL的各分析物单标储备液,用甲醇配制成10 μg/mL混合标准溶液(MeP和BP-3为40 mg/L)。混合稳定同位素内标储备液:按上述相同方法配制混合内标储备液,质量浓度均为0.5 mg/L, 4 ℃冰箱保存。使用时将混合内标储备液用甲醇稀释至0.1 mg/L,配制成混合内标工作液。

标准工作溶液配制:用甲醇将混合标准溶液稀释为2000、1000、500、250、100、50、25、10 μg/L系列标准溶液(MeP和BP-3为8000、4000、2000、1000、400、200、100、40 μg/L)。分别加入50 μL系列标准溶液、75 μL混合内标工作液和375 μL乙腈-水(20∶80, v/v),最终配制为200、100、50、25、10、5、2.5、1 μg/L的标准工作溶液(MeP和BP-3为800、400、200、100、40、20、10、4 μg/L)。

### 1.4 样品前处理

准确移取1 mL尿液到5 mL聚丙烯离心管中。依次加入500 μL乙酸铵-酶缓冲溶液(酶活力相当于500 units/mL)和75 μL混合内标工作液(内标使用量相当于7.5 ng)。充分混匀后在37 ℃下水浴酶解过夜(≥16 h)。酶解结束后取出样品降至室温,在4500 r/min条件下离心10 min,取上清液进行固相萃取。依次用3 mL甲醇和3 mL水活化Oasis HLB固相萃取小柱,将上清液转移至固相萃取柱,在不加压力的条件下依靠自然重力过柱。样本过柱后,用2 mL乙腈-水(25∶75, v/v)快速淋洗,空气抽干3~5 min。4 mL甲醇溶液分2次缓慢洗脱,氮气吹至近干,500 μL乙腈-水(20∶80, v/v)复溶,充分涡旋混匀后过滤膜上机测定。

### 1.5 质量控制

样品检测过程中均添加低、高两个水平的混合内部质控样(IQC)、2个NIST SRM 3672质控样和过程空白样,以保证方法的准确性并控制样品前处理过程中的污染。PCPs极易残留于实验环境中对实验结果产生影响,因此每次实验前后均应对实验环境和装置进行清洁,此外实验过程中尽可能使用玻璃器皿,每批次使用的枪头、离心管等实验耗材均先进行空白评估,保证各分析物无空白引入。

## 2 结果与讨论

### 2.1 液相色谱条件优化

#### 2.1.1 色谱柱的选择

比较了Acquity BEH C_18_柱(100 mm×2.1 mm, 1.7 μm)和Acquity UPLC HSS T_3_柱(100 mm×2.1 mm, 1.8 μm)对12种分析物的色谱分离效果。结果显示,在乙腈-水的流动相体系下,12种分析物在BEH C_18_柱上峰形良好,而经HSS T_3_柱分离后的12种分析物的出峰时间更长,其中PrP、BuP、4-OHBP、BP-1和TCC存在色谱峰拖尾的情况,推测原因如下:HSS T_3_柱是在C_18_键合相的基础上增加了极性基团的修饰,其键合相的比例低于BEH C_18_柱,对极性物质的反向保留能力更强,延长对极性物质的保留时间。因此,在兼顾灵敏度和分离效果的前提下,最终选择BEH C_18_柱作为分离柱。

#### 2.1.2 流动相的选择

确定色谱柱后,用初始流动相配制10 μg/L的混合标准溶液,分别在甲醇-水和乙腈-水两种流动相体系下进样,通过比较峰形和质谱响应确定最优流动相。结果显示,12种分析物在甲醇-水体系下的峰形更好,出峰时间更早,但BP-3在该体系的响应显著低于乙腈-水体系,导致其无法准确定量,通过调整流动相梯度洗脱比例也依然无法改善。进一步比较A相为水,B相分别为甲醇、乙腈和甲醇-乙腈(50∶50, v/v)3种流动相体系下SRM NIST 3672中BP-3的测定结果,发现测定值与参考值的比值分别为0.21、1.04和0.50,可见甲醇对质谱响应有较严重的抑制作用,而在乙腈-水体系中可以实现准确定量。通过分析BP-3的分子结构式我们发现,在ESI^-^模式下,BP-3羟基基团上H^+^解离形成母离子,与甲醇等质子化溶剂电离产生的H^+^结合,使BP-3母离子生成受抑制。而乙腈是非质子化溶剂,属弱质子受体,其中N带孤对电子,可以结合溶剂体系中的电子,促进母离子的生成,因此质谱峰面积响应增大。其他BP类化合物如4-OHBP、BP-1和BP-8等物质也出现此现象,但BP-3的差异更为明显。综合考虑最终选择乙腈-水作为流动相。

当复溶溶剂的强度超过初始流动相就会产生溶剂效应。为避免溶剂效应,通常情况都选择初始流动相作为复溶溶剂。为改善分析物在该流动相体系下的峰形,减小溶剂效应,比较了不同体积分数乙腈水溶液配制的各分析物标准溶液出峰情况。以MeP为例,有机相比例在20%,即为初始流动相时,各分析物峰形得到明显改善,因此最终选择乙腈-水(20∶80, v/v)配制标准溶液并作为复溶溶剂。最优流动相条件下12种分析物的色谱图见图1。

**图1:**
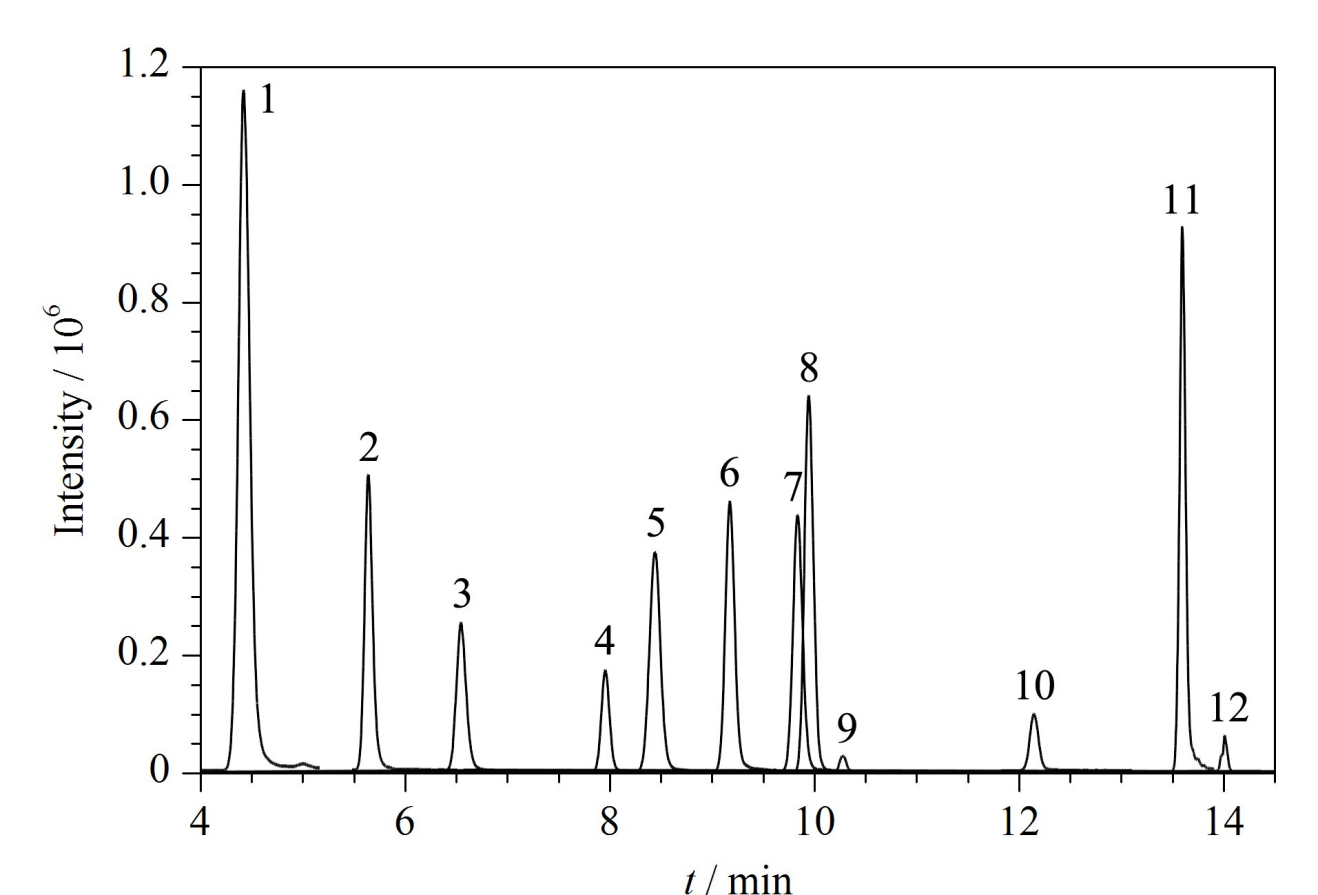
最优流动相条件下12种目标物(10 μg/L)的色谱图 1. MeP; 2. BP-2; 3. EtP; 4. 4-OHBP; 5. PrP; 6. BP-1; 7. BuP; 8. BzP; 9. BP-8; 10. BP-3; 11. TCC; 12. TCS.

### 2.2 前处理条件优化

#### 2.2.1 酶解条件优化

分析物进入人体24 h后,主要以葡萄糖醛酸/硫酸酯结合物形式经尿液排出体外,其中PB约有80.5%~85.3%以4-羟基苯甲酸(4-HB)或结合态存在于尿液中^[27]^,肝脏是BP、TCC和TCS的主要蓄积和代谢器官,经肝脏代谢后其主要以结合物形式经尿液排出^[28⇓-30]^。因此,为准确评估人体内各PCPs的总暴露情况,应先对尿液进行酶解处理以释放结合态的PCPs。为获得最佳酶解效果,分别以NIST SRM 3672和混合尿样作为样品,比较来自罗曼蜗牛和大肠杆菌(*E. coli K*_12_)的*β*-葡萄糖醛酸酶对酶解效果的影响。结果显示,使用来自*Helix pomatia*的*β*-葡萄糖醛酸酶作为水解酶时,NIST SRM 3672和混合尿样中PCPs的测定值最高,而使用来自*E. coli K*_12_的*β*-葡萄糖醛酸酶时,测定值只有前者的50%~80%,进一步优化酶解pH值及增加酶活力也未得到改善,究其原因可能是来自软体动物如*Helix pomatia*的酶也具有硫酸酯酶活性,但*E. coli K*_12_酶不能有效酶解硫酸酯结合态的PCPs。本方法选择罗曼蜗牛*β*-葡萄糖醛酸酶用于样品酶解。

本文采用单因素考察,在控制其余变量一致的前提下分别对pH值(pH=5.0和pH=6.0)、酶活力(100~1000 units/mL)和酶解时间(1、2、4、16和24 h)等因素进行优化。结果发现,pH值对酶解效果影响不大。NIST SRM 3672中6种PCPs在不同酶活力和酶解时间的测定值与参考值之比如图2所示,在控制酶解pH=5.0,酶解时间≥16 h的条件下,当酶活力在400~1000 units/mL时,6种PCPs的测定结果达到平台值,与NIST SRM 3672参考值之比为0.90~1.10,混合尿样的酶解条件与标准物质的一致,且在此条件下过程空白基本稳定,因此,最终确定酶活力为500 units/mL。同样地,控制酶解pH=5.0和酶活力为500 units/mL,确定最优酶解时间为过夜酶解(≥16 h)。

**图2:**
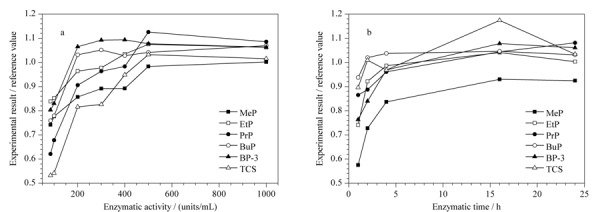
不同(a)酶活力和(b)酶解时间条件下NIST SRM 3672中的6种PCPs测定值与参考值的比值

#### 2.2.2 固相萃取柱的选择

以本底较低的实际尿液为样品,通过评价加标回收率,比较Waters Oasis HLB、Agilent Bond Elut NEXUS和Waters Sep-Pak C_18_ 3种不同固相萃取柱对分析物的净化效果。通过回收各固相萃取柱的上样过滤液,比较其中各分析物的绝对回收率用以评估不同小柱的保留效率,结果显示3种萃取柱对12种分析物保留效率均良好,仅有0.38%~1.60%的MeP、EtP和PrP无法留在柱上。进一步回收淋洗过滤液,结果显示在乙腈-水(25∶75, v/v)的淋洗条件下,Agilent Bond Elut NEXUS和Waters Sep-Pak C_18_柱对部分分析物的保留效果不甚理想,如EtP的绝对回收率为43.2%和15.1%,而Waters Oasis HLB柱可以更好地保留分析物。最后实验得到HLB萃取柱洗脱液中12种分析物的绝对回收率为66.6%~102.5%,经内标校正后的相对回收率为94.3%~112.7%,综合考虑采用Waters Oasis HLB作为固相萃取柱。

#### 2.2.3 淋洗条件优化

通过比较各分析物的色谱峰面积,考察不同体积分数(10%、20%、25%和30%)的甲醇和乙腈水溶液对分析物淋洗效果的影响。结果显示,以乙腈水溶液作为淋洗液时,MeP具有更高的质谱响应信号,其余11种分析物的响应信号与甲醇水溶液无明显区别。为提高分析物的灵敏度,实验选择乙腈水溶液作为淋洗液。通过进一步优化有机相比例发现,当乙腈比例增加时,分析物的定量离子峰面积呈上升趋势,且当有机相比例为25%时达到最优状态。HLB固相萃取柱可以很好地保留非极性至中等极性的化合物,增加淋洗液中有机相的比例可以降低淋洗液的极性,有利于去除尿液基质中的干扰物,降低基质效应。

### 2.3 过程空白考察

PCPs广泛存在于环境介质中,过程空白对实验的影响至关重要。本研究对实验过程中所需的容器、试剂和实验耗材等进行空白筛查。结果显示,甲醇溶液、*β*-葡萄糖醛酸酶、乙酸铵溶液以及移液器枪头易受MeP、EtP、PrP和BP-3的污染(见表3)。对于*β*-葡萄糖醛酸酶中的空白值,通过优化酶活力实现在保证酶解效果的同时尽可能降低空白干扰。对于甲醇和乙酸铵等实验中不可避免使用到的试剂,通过使用MS级试剂可有效降低空白值。不同品牌的离心管,空白差异较大,因此对于离心管等实验耗材均应预先进行空白值评估,无空白值方可使用。值得注意的是,测定样品过程中分析物极易残留在通风橱、固相萃取和氮吹装置等实验环境中。为有效控制环境污染对实验结果的影响,在每批次实验开始前和结束后均使用无水乙醇对实验环境进行清洗。

**表3:** PCPs空白值浓度结果

Compound	Pipette tips				Solvents			Centrifuge tube^b^
	1000 μL^a^	200 μL^a^	Dispenser tip^b^		MeOH^c^	NH_4_AC buffer^c^	Enzyme^d^	
MeP	/	1.69	2.02		/	/	2.83	/
EtP	1.56	2.61	3.07		/	/	1.04	0.91
PrP	0.55	1.19	1.28		/	/	0.57	/
BP-3	1.36	3.11	2.83		0.34	0.51	1.42	0.80

a. repeatedly aspirate methanol solution three times and machine determine; b. 5 mL methanol solution soak overnight and machine determine; c. machine determine directly; d. dilute *β*-glucuronidase 20 times with ultrapure water and machine determine; /: not detected.

### 2.4 基质效应评价

选用5个不同来源的尿液,按前处理方法进行处理后,以该5个尿液洗脱液为基质配制1、2.5、5、10、25、50、100、200 μg/L (MeP和BP-3为4、10、20、40、100、200、400、800 μg/L)的尿液基质标准曲线,同时配制相同浓度的甲醇基质标准曲线。按照文献^[31]^的方法,通过比较两个曲线斜率的比值评估基质效应(见图3)。当ME为80%~120%时,判断为弱基质效应;当ME为50%~80%或120%~150%时,判断为中等基质效应;当ME<50%或>150%时,判断为强基质效应。结果显示,部分尿液中MeP、EtP和BP-2表现为强基质效应(26.7%~235.7%), PrP表现为中等基质效应(79.2%~112.0%),其他8种分析物在5种不同来源尿液中的基质效应均较弱(83.3%~115.5%)。采用稳定同位素内标法校正后,12种分析物的基质效应均表现为弱基质效应(91.9%~110.1%)。

**图3:**
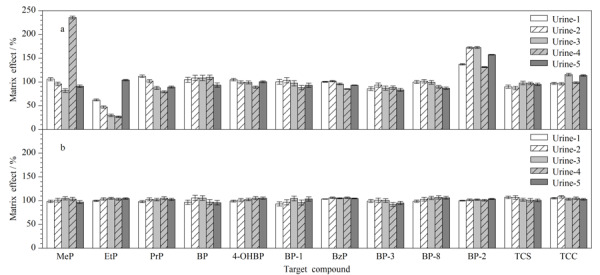
12种PCPs采用稳定同位素内标法(a)校正前和(b)校正后的基质效应(*n*=6)

### 2.5 方法学评价

#### 2.5.1 线性范围、检出限与定量限

配制1、2.5、5、10、25、50、100、200 μg/L(MeP和BP-3为4、10、20、40、100、200、400、800 μg/L)的系列标准溶液,在上述色谱条件和质谱条件下进行测定。以分析物与相应内标的定量离子峰面积之比为纵坐标,以样品中分析物的质量浓度为横坐标绘制标准曲线,12种分析物在其线性范围内的相关系数(*r*)均大于0.999。按照美国环保署(USEPA)检出限测定程序第2版(EPA 821-R-16-006规范文件)^[32]^计算方法检出限(MDL)和方法定量限(MQL)。由于12种典型PCPs均存在过程空白干扰,因此采用7次过程空白的均值+3倍标准差( $\overset{-}{X}$ +3SD)计算MDL, 7次过程空白的 $\overset{-}{X}$ +10SD计算MQL。12种分析物的线性范围、相关系数、MDL和MQL见表4。

**表4:** 12种目标物的线性范围、相关系数、方法检出限和方法定量限

Compound	Linear range/ (μg/L)	*r*	MDL/ (μg/L)	MQL/ (μg/L)
MeP	4.00-800	0.9995	0.94	1.15
EtP	1.00-200	0.9998	0.51	0.60
PrP	1.00-200	0.9996	0.43	0.76
BuP	1.00-200	0.9998	0.22	0.24
BzP	1.00-200	0.9998	0.19	0.20
BP-1	1.00-200	0.9994	0.06	0.08
BP-2	1.00-200	0.9994	0.12	0.12
BP-3	4.00-800	0.9998	0.49	0.70
BP-8	1.00-200	0.9997	0.36	0.39
4-OHBP	1.00-200	0.9994	0.13	0.21
TCC	1.00-200	0.9992	0.32	0.75
TCS	5.00-200	0.9992	1.09	3.63

#### 2.5.2 回收率与精密度

选择本底较低的实际尿样进行加标回收试验,12种分析物低浓度均采用MQL加标,中、高加标水平MeP和BP-3为40 μg/L和200 μg/L,其余分析物为10 μg/L和50 μg/L,在同一自然日每个水平进行6次平行试验,计算加标回收率和日内精密度。在不同自然日分6次进行测定,计算日间精密度。结果如表5所示。

**表5:** 12种目标物的加标回收率、日间精密度和日内精密度(*n*=6)

Compound	Low			Medium			High		Inter-day RSD/%	Intra-day RSD/%
	Recovery/%	RSD/%		Recovery/%	RSD/%		Recovery/%	RSD/%		
MeP	93.2	2.2		100.7	2.1		107.3	3.4	4.9	2.0
EtP	95.4	4.4		97.8	2.3		104.4	1.5	3.7	2.7
PrP	97.1	1.3		97.1	1.3		103.5	4.6	4.4	4.0
BuP	94.7	4.2		100.3	2.0		101.8	2.9	3.8	3.5
BzP	103.5	2.9		104.9	2.3		108.0	3.7	4.2	3.9
BP-1	89.5	4.1		99.5	1.1		101.4	1.2	4.2	3.3
BP-2	99.7	2.3		105.3	3.9		97.1	1.5	3.9	5.2
BP-3	89.8	2.6		91.0	2.4		99.0	1.2	7.1	2.1
BP-8	105.0	8.5		101.4	2.8		103.9	4.5	7.1	4.9
4-OHBP	100.2	3.0		102.8	3.0		106.3	3.2	3.7	5.1
TCC	94.8	7.4		99.9	3.2		100.7	2.1	4.3	5.5
TCS	111.8	7.1		95.1	12.8		98.0	7.1	8.9	10.6

### 2.6 正确度评价

每批次样品测定时,均设置NIST SRM 3672和IQC以评估方法的正确度,由于NIST SRM 3672认证证书中仅包含6种分析物,因此设置IQC共同评估。本方法NIST SRM 3672测定结果与已有文献报道^[33,34]^结果相当,且与证书参考值比对结果在可接受范围内,7次测定的批间精密度为1.2%~4.0%(见表6), 7次IQC测定结果(见图4)均在 $\overset{-}{X}$ ±2SD范围内(仅一批次4-OHBP除外),说明本方法正确度满足分析要求,可以用于实际人体尿液样品中分析物的测定。

**表6:** NIST SRM 3672测定值与参考值比较(*n*=7)

Compound	Reference value/(μg/kg)		Experimental result/(μg/kg)	RSD/%
MeP	113±	2	102-110	3.1
EtP	8.1±	0.2	7.90-8.27	1.8
PrP	17.6±	0.3	16.6-17.7	2.3
BuP	11.1±	0.2	10.7-11.1	1.2
BP-3	191±	5	191-209	3.5
TCS	17.7±	0.5	15.1-16.9	4.0

**图4:**
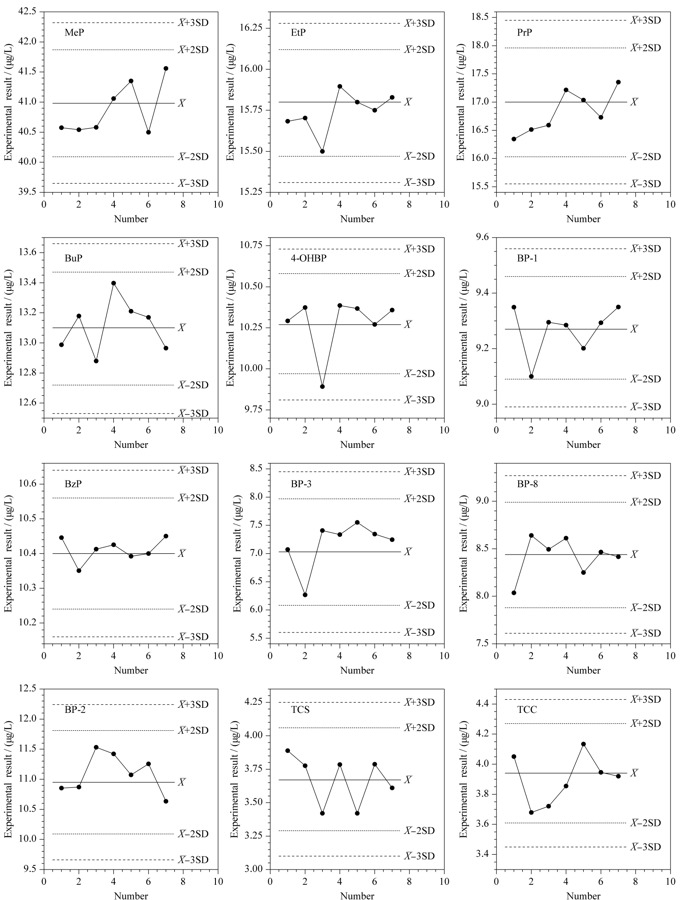
内部质控样正确度评价结果

### 2.7 实际样品测定

收集某地区127份健康人群尿液样本并采用本方法对其进行检测,其中本研究经伦理审查委员会评审同意,志愿者均签署知情同意书。结果如表7所示,除BzP和BP-8未检出外,其余10种典型PCPs均有检出,总体检出率为1.7%~99.7%,其中MeP、EtP和PrP检出率显著高于其他分析物。MeP、EtP和PrP检出含量较高,中位值分别为13.3、1.04和0.83 μg/L。此外,值得注意的是,个别人群存在BP-3高暴露,其尿样含量可达1.14×10^3^ μg/L。

**表7:** 12种典型PCPs在人尿中的测定值

Compound	DF/%	Contents/(μg/L)				
		P_5_	P_25_	P_50_	P_75_	P_95_
MeP	99.7	2.10	5.72	13.3	51.5	347
EtP	66.1	ND	ND	1.04	5.37	80.3
PrP	58.2	ND	ND	0.83	18.0	125
BuP	7.9	ND	ND	ND	ND	0.61
BzP	0.0	ND	ND	ND	ND	ND
BP-1	39.7	ND	ND	ND	0.18	4.60
BP-2	1.7	ND	ND	ND	ND	ND
BP-3	35.3	ND	ND	ND	0.71	18.1
BP-8	0.0	ND	ND	ND	ND	ND
4-OHBP	4.4	ND	ND	ND	ND	ND
TCC	5.1	ND	ND	ND	ND	0.54
TCS	13.7	ND	ND	ND	ND	15.6

DF: detection frequency; P_5_, P_25_, P_50_, P_75_ and P_95_: the mass concentrations of targeted analytes of 5th, 25th, 50th, 75th and 95th percentile; ND: no detectable.

### 2.8 方法比较

通过调研文献我们发现,目前能够同时测定包括防腐剂、紫外吸收剂和抗菌剂在内的PCPs的方法有限。如表8所示,我们对采用SPE-LC-MS/MS的同类研究进行比较,发现本方法的MDL处于较低水平,其中文献[36]的MDL(0.05 μg/L)显著低于其他方法,这是由于该方法是按照3倍信噪比(*S/N*)进行计算的。其次,尿液样本量是检测方法优化过程中的核心问题之一,表8中文献方法的样本量在1.0~4.0 mL范围内,本方法仅需1.0 mL样本量且浓缩倍数为1倍。此外,美国疾病预防控制中心^[38]^采用Online SPE-HPLC-MS/MS方法开展生物监测,所需样本量虽仅需0.1 mL,但MDL相对较高,仪器普及性不高,国内不易推广。综合考虑,本方法采用UPLC-MS/MS,在保证方法灵敏度的同时减少对于生物样本的消耗,适用于开展环境健康研究中人群尿液样本PCPs的生物监测,同时也可为PCPs暴露的健康风险评估提供可靠的技术支持。

**表8:** 本方法与文献报道的人尿中PCPs检测方法的比较

No.	Target compounds				Urine sample/mL	Extraction method	Instrumental analysis method	MDL/ (μg/L)	Ref.
	Paraben	Benzophenone	Antiseptic	n					
1	√	√	/	12	3.0	SPE	UPLC-MS/MS	≤1.23	[35]
2	√	/	√	8	4.0	SPE	LC-MS/MS	≤0.05	[36]
3	√	/	√	13	1.0	SPE	HPLC-MS/MS	≤1.0	[37]
4	√	√	√	8	0.1	On-line SPE	HPLC-MS/MS	≤2.0	[38]
5	√	√	√	12	1.0	SPE	UPLC-MS/MS	≤1.0	this study

*n*: the total number of the target compounds in the method; /: not mentioned.

## 3 结论

本研究建立了人尿中12种典型PCPs的UPLC-MS/MS检测方法。相比于现有研究,本方法能够同时测定包括防腐剂、紫外吸收剂和抗菌剂在内的12种PCPs,操作简便、定量准确、重复性好,可应用于人群中典型PCPs类物质的内暴露检测,为我国开展人群生物监测项目奠定基础。
